# 

**Published:** 1872-10

**Authors:** 


### Books and Pamphlets Received.

The Principles and Practice of Surgery. By Frank Hastings Hamilton, A. M., M. D., LL. D., etc. Illustrated with 467 Engravings on Wood. New York: William Wood & Co., 1872. Buffalo: T. Butler & Son.

Ovarian Tumors: Their Pathology, Diagnosis, and Treatment, especially by Ovariotomy. By E. Randolph Peaselee, M. D., LL. D., etc. With fifty-six illustrations on wood. New York: D. Appleton & Co., 1872. Buffalo: Martin Taylor.

The Science and Practice of Medicine. By William Aitken, M. D., etc. The Third American from the Sixth London Edition. Edited with additions. By Meredith Clymer, M. D. In two vols. With numerous engravings. Philadelphia. Lindsay & Blakiston, 1872. Buffalo: T. Butler & Son.

A Treatise on Diseases of the Nervous System. By William A. Hammond, M. D., etc. Third edition, with additions and corrections. New York: D. Appleton & Co., 1872. Buffalo: Martin Taylor.

On the Functional Diseases of the Renal, Urinary and Reproductive Organs, with a general review of Urinary Pathology. By D. Campbell Black, M. D., L. R. C. S. Edin., etc. Philadelphia: Lindsay & Blakiston, 1872. Buffalo; T. Butler & Son.

The Treatment of Syphilis by Subcutaneous Sublimate Injections. By Dr. George Lewin. Berlin. Translated by Carl Proegler, M. D., and E. H. Gale, M. D. Philadelphia: Lindsay & Blakiston, 1872. Buffalo: T. Butler & Son.

Epidemic Cerebro-Spinal Meningitis. With an Appendix on some points on the cause of the disease as shown by the History of the present Epidemic in the City of New York. By Meredith Clymer, M. D. Philadelphia: Lindsay & Blakiston, 1872. Buffalo. T. Butler & Son.

Small-Pox: The Predisposing Conditions and their Preventives. With a Scientific Exposition of Vaccination. By Dr. Carl Both. Second Edition. Boston: Alex. Moore, 1872. Buffalo: Martin Taylor.

Norman Ovariotomy. By Robert Battey, M. D., Rome, Ga.

Transactions of the Georgia Medical Association at its Twenty-Third Annual Meeting, held at Columbus, Ga., April, 1872.

Re-introduction of Ether into England. Read at the Suffolk District Medical Society. By B. Joy Jeffries, M. D. Re-printed from the Boston Medical Journal, of October 3d, 1872.

The Physiology of the Brain. A Discourse delivered before the Oneida County Society, July 9th, 1872. By C. B. Coventry, M. D.

Facts of Vital Statistics in the United States; with Tables and Diagrams. Extracts from an Address by J. M. Toner, M. D.

### Medical and Surgical Journal

PUBLISHED MONTHLY,

Containing Original Articles, Reports of Medical Societies and Hospitals, Editorial, Reviews, Correspondence, Army News, etc., etc.; including the usual variety of Medical Periodical Publications. Specimen copies sent on application.

Address Buffalo Medical & Surgical Journal, Buffalo, N. Y.

TERMS OF SUBSCRIPTION, - - $3.00 PER YEAR, IN ADVANCE.

